# RE: Evaluation of sexual function in patients submitted to ureteroscopic procedures

**DOI:** 10.1590/S1677-5538.IBJU.2016.0066

**Published:** 2016

**Authors:** Mustafa Kadihasanoglu, Emin Özbek

**Affiliations:** 1 Department of Urology, Istanbul Training and Research Hospital, Istanbul Turkey

*Dear Editor,*

We have read the article by Eryildirim et al. (1) which was interesting study evaluating the possible effects of ureteroscopy on the male and female sexual function. In this study they tried to evaluate the male and female sexual function before and after ureteroscopic procedure. They showed that the pre-and postoperative International Index of Erectile Function scores and Female Sexual Function Index scores were not influenced by ureteroscopy.

Multifactorial nature of sexuality should be considered during the evaluation of the sexual function of men and women. The factors affecting female sexual function include genetics, mental health status, symptoms of depression and anxiety, quality of relationships, menopause, hormonal imbalance, hysterectomy, ovariectomy, sexual abuse, negative sexual attitude, negative body image, drug and alcohol abuse, sexual orientation, childbirth and its outcomes, mode of delivery, number of childbirths, breastfeeding, and fears of pregnancy or sexually transmitted diseases (2). Similarly, male sexual dysfunction can result from physiological causes including depression, anxiety, stress, other mental health problems and physical causes including diabetes, obesity, metabolic syndrome, cardiovascular diseases, hypertension, treatments for prostate cancer, benign prostate hyperplasia, neurological diseases, hypogonadism, smoking, and pelvic surgeries (3). In addition to all well-known factors, urolithiasis and erectile dysfunction are defined as systemic diseases which are associated hormonal and metabolic disorders such as insulin resistance, obesity and metabolic syndrome (3, 4).

Thus, we consider that these factors for male and female sexual dysfunction, as mentioned above, are limitations of this study, because the authors did not exclude the patients with these factors and they aimed to reach a conclusion that ureteroscopy can adverse effect on male sexual function. Moreover, they did not suggest any pathophysiological mechanism resulted from ureteroscopy for sexual dysfunction. If the authors considered that the anxiety and pain may be a cause of sexual dysfunction, they should be evaluated with valid scales and evaluation forms. In addition, the authors did not report that the patient use any medical treatment including hormones, and phosphodiesterase inhibitors before and after treatment, because these medications can affect the sexual function. Furthermore, the interval for the postoperative evaluation of sexual dysfunction was only 4 weeks. Sofer et al suggested that complete recovery of sexual function is found at the 3 month follow-up of all men who had normal of slightly impaired sexual function before going an endourological procedure (5). As a result, we claim that these factors should be indicated as a limitation to strengthen the outcomes of the study.

*Mustafa Kadihasanoglu, MD* *Department of Urology, Istanbul Training and Research Hospital, Istanbul Turkey* *E-mail: kadihasanoglu@gmail.com*

## References

[1] Eryildirim B, Tuncer M, Sahin C, Yucetas U, Sarica K (2015). Evaluation of sexual function in patients submitted to ureteroscopic procedures. Int Braz J Urol.

[2] Khajehei M, Doherty M, Tilley PJ (2015). An update on sexual function and dysfunction in women. Arch Womens Ment Health.

[3] Dean RC, Lue TF (2005). Physiology of penile erection and pathophysiology of erectile dysfunction. Urol Clin North Am.

[4] Otunctemur A, Ozbek E, Cakir SS, Dursun M, Polat EC, Ozcan L (2014). Association of erectile dysfunction and urolithiasis. Arch Ital Urol Androl.

[5] Sofer M, Yehiely R, Greenstein A, Bar-Yosef Y, Matzkin H, Chen J (2012). Endourological procedures and sexual dysfunction: a prospective multivariate analysis. BJU Int.

